# First trimester screening of circulating C19MC microRNAs and the evaluation of their potential to predict the onset of preeclampsia and IUGR

**DOI:** 10.1371/journal.pone.0171756

**Published:** 2017-02-09

**Authors:** Ilona Hromadnikova, Katerina Kotlabova, Katarina Ivankova, Ladislav Krofta

**Affiliations:** 1 Department of Molecular Biology and Cell Pathology, Third Faculty of Medicine, Charles University, Prague, Czech Republic; 2 Institute for the Care of the Mother and Child, Third Faculty of Medicine, Charles University, Prague, Czech Republic; Academic Medical Centre, University of Amsterdam, NETHERLANDS

## Abstract

**Objectives:**

A nested case control study of a longitudinal cohort comparing pregnant women enrolled at 10 to 13 gestational weeks was carried out to evaluate risk assessment for preeclampsia and IUGR based on circulating placental specific C19MC microRNAs in early pregnancy.

**Methods:**

The expression of placental specific C19MC microRNAs (miR-516b-5p, miR-517-5p, miR-518b, miR-520a-5p, miR-520h, and miR-525-5p) was determined in plasma samples from pregnancies that subsequently developed preeclampsia (n = 21), IUGR (n = 18), and 58 normal pregnancies using real-time PCR and comparative Ct method relative to synthetic *Caenorhabditis elegans* microRNA (cel-miR-39).

**Results:**

Circulating C19MC microRNAs were up-regulated (miR-517-5p, p = 0.005; miR-518b, p = 0.013; miR-520h, p = 0.021) or showed a trend toward up-regulation in patients destined to develop preeclampsia (miR-520a-5p, p = 0.067; miR-525-5p, p = 0.073). MiR-517-5p had the best predictive performance for preeclampsia with a sensitivity of 42.9%, a specificity of 86.2%, a PPV of 52.9% and a NPV of 80.6%. The combination of all examined circulating C19MC microRNAs had no advantage over using only the miR-517-5p biomarker to predict the occurrence of preeclampsia (a sensitivity of 20.6%, a specificity of 90.8%, a PPV of 44.8%, and a NPV of 76.0%).

**Conclusions:**

Up-regulation of miR-517-5p, miR-518b and miR-520h was associated with a risk of later development of preeclampsia. First trimester screening of extracellular miR-517-5p identified a proportion of women with subsequent preeclampsia. No circulating C19MC microRNA biomarkers were identified that could predict later occurrence of IUGR.

## Introduction

MicroRNAs belong to a family of small noncoding RNAs that regulate gene expression at the post-transcriptional level by degrading or blocking translation of messenger RNA (mRNA) targets [1, 2]. The diagnostic potential of specific molecular biomarkers and their implementation in predictive and diagnostic algorithms for pregnancy related complications are subjects of considerable interest [3].

Although the study by Luque et al. [4] demonstrated that maternal serum microRNA assessment at the end of the first trimester of pregnancy did not appear to have any predictive value for early preeclampsia (requiring delivery before 34 weeks), the data from other studies strongly supported the need for a more detailed exploration of microRNAs in maternal circulation with the view toward routine assessment in everyday practice, and recognition that they represent potential biomarkers for pregnancy related complications [5–8].

Our pilot study suggested the potential of using circulating C19MC microRNAs (miR-520h, miR-518b, miR-516b-5p, and miR-517-5p) to differentiate, at the beginning of gestation (weeks 10–13), between patients that will develop gestational hypertension and those that will have normal pregnancies [9]. First trimester screening of extracellular miR-520h alone or in combination with miR-518b was able to identify a significant proportion of women that went on to develop gestational hypertension [9].

Recent studies of Ura et al. [5] have shown that severe preeclampsia is associated with alterations in extracellular microRNA expression (miR-1233, miR-520, miR-210 and miR-144) during the early stages of gestation (12–14 weeks).

Latest preliminary studies by Winger et al. [6, 10] demonstrated that profiling of selected microRNA biomarkers in maternal peripheral blood mononuclear cells, prior the end of the first trimester, may successfully predict adverse outcomes such as preeclampsia and miscarriage. In addition, microRNA quantification of maternal blood cells was also able to predict the occurrence of late preeclampsia.

As far as we know, this is the first study using first trimester screening of women at risk of developing preeclampsia or IUGR relative to circulating C19MC microRNAs (miR-516b-5p, miR-517-5p, miR-518b, miR-520a-5p, miR-520h, and miR-525-5p). Here, we discuss for the first time the effectiveness of circulating C19MC microRNAs as predictors of preeclampsia and IUGR. This study is a part of a long-term research program focused on the description of the complex pathogenesis mechanisms involved in pregnancy-related complications, with the goal of identifying of novel biomarkers that can diagnose and/or predict pregnancy-related complications [8, 9, 11].

## Materials and methods

### Patients

The study was retrospective, designed to run from 2012–2016. The study cohort consisted of 1464 consecutive Caucasian singleton pregnant women undergoing first trimester screening at 10–13 gestational weeks. Of 1464 pregnant women participating in first trimester screening, 359 were lost to follow-up (they underwent first trimester screening at the Center of Prenatal Diagnosis in our health care facility, but delivered at another health care facility), 21 developed preeclampsia (1.9%) and 18 pregnancies (1.63%) were complicated by intrauterine growth restriction (IUGR). The clinical characteristics of normal and complicated pregnancies are presented in Table 1 and also provided as a supplementary table in Excel format (S1 Table).

**Table 1:** Maternal and neonatal characteristics of normal and complicated pregnancies.

	Normal pregnancies	Preeclampsia	IUGR	p-value^1^	p-value^2^	p-value^3^
	(n = 58)	(n = 21)	(n = 18)			
Maternal age (years)	32.71±0.49 (27–42)	34.33±1.13 (27–42)	34.56±1.08 (27–43)	0.145	0.127	0.085
GA at sampling (weeks)	10.86±0.12 (10–13)	11.18±0.31 (10–13.56)	10.58±0.17 (10–13.28)	0.191	0.244	0.250
GA at delivery (weeks)	39.91±0.16 (38–41.56)	35.0±1.05 (21.42–40.7)	36.21±0.62 (28.28–39.0)	**<0.001**	**<0.001**	**<0.001**
Mode of delivery						
● Vaginal	54 (93.1%)	2 (9.5%)	3 (16.7%)	**<0.001**	**<0.001**	**<0.001**
● Cesarean section	4 (6.9%)	19 (90.5%)	15 (83.3%)			
Fetal birth weight (g)	3450.7±66.62 (2930–4340)	2627.5±205.93 (930–3860)	2079.6±127.48 (746–2840)	**<0.001**	**<0.001**	**<0.001**
Fetal sex						
● Boy	30 (51.7%)	12 (57.2%)	7 (38.9%)	0.517	0.669	0.341
● Girl	28 (48.3%)	9 (42.8%)	11 (61.1%)			
Primiparity						
● Yes	28 (48.3%)	14 (66.7%)	12 (66.7%)	0.209	0.147	0.172
● No	30 (51.7%)	7 (33.3%)	6 (33.3%)			

Data are presented as mean±SE (range) for continuous variables and as number (percent) for categorical variables.

Statistically significant results are marked in bold.

p-value^1^: the comparison among three groups.

Continuous variables were compared using ANOVA. Categorical variables were compared using Fisher´s exact test.

p-value^2^: the comparison among preeclampsia and normal pregnancies.

p-value^3^: the comparison among IUGR and normal pregnancies.

Continuous variables were compared using the t-test. Categorical variables were compared using a chi-square test.; GA, gestational age.

The case cohort included all 21 preeclamptic pregnancies, 18 IUGR pregnancies, and the control cohort (58 normal pregnancies), which were chosen based on equal blood sample storage times and gestational age. Of the 21 patients with preeclampsia, 7 had symptoms of mild preeclampsia and 14 were diagnosed with severe preeclampsia. Six preeclamptic patients required delivery before 34 weeks of gestation and 15 patients delivered after 34 weeks of gestation. Preeclampsia occurred both in previously normotensive patients (18 cases), or was superimposed on pre-existing hypertension (3 cases).

Three growth-retarded fetuses were delivered before 34 weeks of gestation and 15 after 34 weeks of gestation. Oligohydramnios or anhydramnios were present in 2 growth-restricted fetuses. The cerebro-placental ratio (CPR), expressed as a ratio between the middle cerebral artery and the umbilical artery pulsatility indexes was below the fifth percentile in 11 IUGR cases. Absent or reversed end-diastolic velocity waveforms in the umbilical artery occurred in 1 IUGR case.

Preeclampsia was defined as blood pressure > 140/90 mmHg on two determinations 4 hours apart that was associated with proteinuria > 300 mg/24 h after 20 weeks of gestation [12]. Severe preeclampsia was diagnosed by the presence of one or more of the following findings: 1) a systolic blood pressure > 160 mmHg or a diastolic blood pressure > 110 mmHg, 2) proteinuria greater than 5 g of protein in a 24-hour sample, 3) very low urine output (less than 500 ml in 24 h), 4) signs of respiratory problems (pulmonary edema or cyanosis), 5) impaired liver functions, 6) signs of central nervous system problems (severe headache, visual disturbances), 7) pain in the epigastric area or right upper quadrant, 8) thrombocytopenia, and 9) the presence of severe fetal growth restriction [12].

Fetal growth restriction was diagnosed when the estimated fetal weight (EFW), calculated using the Hadlock formula (Astraia Software GmbH), was below the tenth percentile for the evaluated gestational age, adjustments were made for the population standards of the Czech Republic. In addition to fetal weight below the threshold of the 10^th^ percentile, IUGR fetuses had at least one of the following pathological finding: an abnormal pulsatility index in the umbilical artery, absent or reversed end-diastolic velocity waveforms in the umbilical artery, an abnormal pulsatility index in the middle cerebral artery, a sign of a blood flow centralization, and a deficiency of amniotic fluid (anhydramnios and oligohydramnios).

Centralization of the fetal circulation represents a protective reaction of the fetus against hypoxia that manifests itself in redistribution of the circulation to the brain, liver, and heart at the expense of the flow reduction in the periphery [13, 14]. The cerebroplacental ratio (CPR) quantifies redistribution of cardiac output by dividing Doppler indices from representative cerebral and fetoplacental vessels.

Normal pregnancies were defined as those without complications that delivered full term, healthy infants weighting > 2500 g after 37 completed weeks of gestation.

All patients provided written informed consent. The study was approved by the Ethics Committee of the Third Faculty of Medicine, Charles University. Gestational age was assessed using ultrasonography between weeks 10–13 weeks plus 6 days.

### Processing of samples

Nine milliliters of peripheral blood were collected into EDTA tubes and centrifuged twice at 1200 g for 10 min at room temperature. Plasma samples were stored at −80°C until subsequent processing.

Total RNA was extracted from 1 mL of plasma and 25 mg of normal placental tissue preserved in RNAlater (Ambion, Austin, USA), followed by an enrichment procedure for small RNAs using a mirVana microRNA Isolation kit (Ambion, Austin, USA). Trizol LS reagent was used in plasma samples for total RNA extraction from biological fluids (Invitrogen, Carlsbad, USA) and preceded the small RNAs enrichment procedure. To minimize DNA contamination, we treated the eluted RNA with 5 μL of DNase I (Fermentas International, Ontario, Canada) for 30 min at 37°C.

### Reverse transcriptase reaction

Each microRNA was reverse transcribed into complementary DNA using TaqMan MicroRNA Assay, containing microRNA-specific stem-loop RT primers, and a TaqMan MicroRNA Reverse Transcription Kit (Applied Biosystems, Branchburg, USA) in a total reaction volume of 50 μL on a 7500 Real-Time PCR system (Applied Biosystems, Branchburg, USA) with the following thermal cycling parameters: 30 minutes at 16°C, 30 minutes at 42°C, 5 minutes at 85°C, and then held at 4°C.

### Quantification of microRNAs

15 μL of cDNA corresponding to each microRNA was mixed with components of TaqMan MicroRNA Assay, and the ingredients of a TaqMan Universal PCR Master Mix (Applied Biosystems, Branchburg, USA) in a total reaction volume of 35 μL. TaqMan PCR conditions were set as described in the TaqMan guidelines. The analysis was performed using a 7500 Real-Time PCR System. All PCRs were performed in duplicates. A sample was considered positive if the amplification signal occurred before the 40^th^ threshold cycle. The characteristics of studied C19MC microRNAs are outlined in Table 2.

**Table 2:** Characteristics of selected C19MC microRNAs.

Assay name	miRBase ID	NCBI Location Chromosome	microRNA sequence	Expression in placenta
hsa-miR-516-5p	hsa-miR-516b-5p	Chr.19: 58920508–58920592 [+]	5´-CAUCUGGAGGUAAGAAGCACUUU-3´	exclusively expressed
hsa-miR-517*	hsa-miR-517-5p	Chr.19: 54215522–54215608 [+]	5´-CCUCUAGAUGGAAGCACUGUCU-3´	high expression
hsa-miR-518b	hsa-miR-518b	Chr.19: 54205991–54206073 [+]	5´-CAAAGCGCUCCCCUUUAGAGGU-3´	exclusively expressed
hsa-miR-520a*	hsa-miR-520a-5p	Chr.19: 54194135–54194219 [+]	5´-CUCCAGAGGGAAGUACUUUCU-3´	high expression
hsa-miR-520h	hsa-miR-520h	Chr.19: 54245766–54245853 [+]	5´-ACAAAGUGCUUCCCUUUAGAGU-3´	exclusively expressed
hsa-miR-525	hsa-miR-525-5p	Chr.19: 54200787–54200871 [+]	5´-CUCCAGAGGGAUGCACUUUCU-3´	exclusively expressed

C19MC microRNAs were divided into two categories (microRNAs exclusively expressed in the placental tissue and those with high expression in the placental tissue) based on information in miRNAMap 2.0 database (http://mirnamap.mbc.nctu.edu.tw/index.php), where the Q-PCR experiments for monitoring the expression profiles of 224 human miRNAs in eighteen major normal tissues in humans are provided. For example, we indicated miR-516b-5p and miR-518b as those to be exclusively expressed in the placental tissue, since according to the miRNAMap 2.0 database miR-516b-5p was shown to be expressed only in the placental tissue and miR-518b to be highly expressed in the placental tissue and rarely expressed in testes. On the other hand, for instance miR-520a-5p showed besides high expression in the placental tissue also low expression in other human tissues involving adipose, bladder, brain, cervix, heart, kidney, liver, lung, muscle, ovary, prostate, small intestine, spleen, testes, thymus, thyroid and trachea.

The expression of particular microRNA in maternal plasma was determined using the comparative Ct method [15] relative to the expression of the same microRNA in a reference sample. A RNA fraction, highly enriched for small RNA, isolated from the fetal part of one randomly selected placenta derived from gestation with normal course (the part of the placenta derived from the chorionic sac that encloses the embryo, consisting of the chorionic plate and villi) was used as a reference sample for relative quantification throughout the study.

Synthetic *C*. *elegans* microRNA (cel-miR-39, Qiagen, Hilden, Germany) was used as an internal control for variations during the preparation of RNA, cDNA synthesis, and real-time PCR. Due to a lack of generally accepted standards, all experimental real-time qRT-PCR data were normalized to cel-miR-39, since it shows no sequence homology to any human microRNA. 1 μl of 0.1 nM cel-miR-39 was spiked in after incubation with Trizol LS reagent to human plasma and reference samples. The following equation was used to compare gene expression between various samples:

$$2^{-△△Ct}=\left[\left(CtparticularC19MCmicroRNA-Ctcel-miR-39\right)testedsample-\left(CtparticularC19MCmicroRNA-Ctcel-miR-39\right)referencesample\right]$$

### Statistical analysis

Data normality was assessed using the Shapiro-Wilk test, which showed that our clinical data (maternal age) followed a normal distribution. Therefore, microRNA levels were compared between groups using the parametric test (*t*-test) with Statistica software (version 9.0; StatSoft, Inc., USA). Since the Bonferroni correction was used to address the problem of multiple comparisons, the significance level was established at p < 0.025.

Receivers operating characteristic (ROC) curves were constructed to calculate the area under the curve (AUC) and the best cut-off point for particular placental specific microRNA was used in order to calculate the respective sensitivity, specificity, predictive values, and likelihood ratios for prediction of preeclampsia.

Data analysis was performed, and box plots were generated using Statistica software (version 9.0; StatSoft, Inc., USA). Each box encompasses the mean (dark horizontal line) of normalized gene expression values for microRNAs of interest in cohorts, one standard error above and below the mean in the box, and the 95% confidence interval are shown as bars (standard deviation). Outliers are indicated by circles, and extremes are indicated by asterisk.

## Results

### Up-regulation of circulating C19MC microRNAs in pregnancies that developed preeclampsia

**Fig 1:**
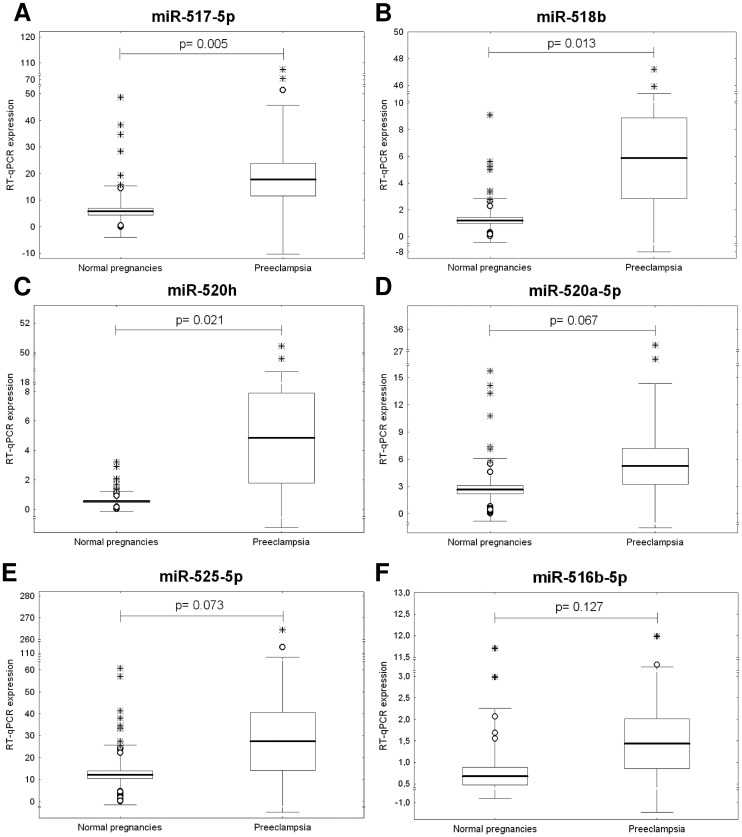
Up-regulation of circulating C19MC microRNAs in pregnancies destined to developed preeclampsia.

Overall, increased levels of **miR-517-5p** (mean 17.770 ± 6.107 vs. 5.713 ± 1.271, p = 0.005), **miR-518b** (mean 5.872 ± 3.024 vs. 1.204 ± 0.217, p = 0.013), and **miR-520h** (mean 4.826 ± 3.050 vs. 0.542 ± 0.088, p = 0.021) were observed during the first trimester of gestation in maternal plasma samples derived from the women who developed preeclampsia compared to women with normal pregnancies. Simultaneously, a trend towards increased plasma levels of **miR-520a-5p** (mean 5.227 ± 1.984 vs. 2.628 ± 0.452, p = 0.067), **miR-525-5p** (mean 27.365 ± 13.186 vs. 12.136 ± 1.786, p = 0.073), and **miR-516b-5p** (mean 1.431 ± 0.580 vs. 0.678 ± 0.207, p = 0.127) in patients destined to develop preeclampsia was identified (Fig 1).

### First trimester screening of circulating C19MC microRNAs does not differentiate between pregnancies with later onset of IUGR and pregnancies with normal course of gestation

No difference in plasma levels of **miR-516b-5p** (mean 0.678 ± 0.207 vs. 0.515 ± 0.205, p = 0.694), **miR-517-5p** (mean 5.713 ± 1.271 vs. 5.674 ± 0.948, p = 0.932), **miR-518b** (mean 1.204 ± 0.217 vs. 0.975 ± 0.393, p = 0.606), **miR-520a-5p** (mean 2.628 ± 0.452 vs. 2.262 ± 1.116, p = 0.809), **miR-520h** (mean 0.542 ± 0.088 vs. 0.317 ± 0.125, p = 0.242), and **miR-525-5p** (mean 12.136 ± 1.786 vs. 7.746 ± 2.872, p = 0.210) between the control group and the group of patients destined to develop IUGR was found.

### First trimester screening of circulating C19MC microRNAs in the identification of preeclampsia pregnancies

**Fig 2:**
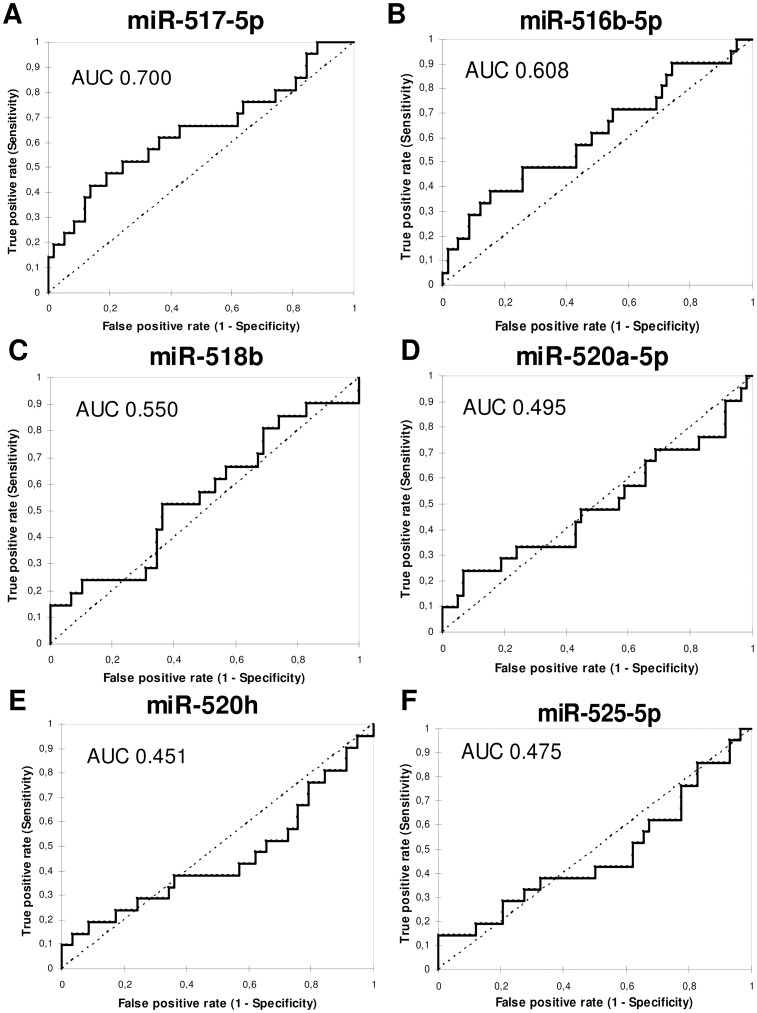
Receiver operating characteristic curves for prediction of development of preeclampsia.

First, the predictive accuracy of single first trimester plasma microRNA biomarkers for preeclampsia was assessed. The largest area under the curve (AUC) was observed for **miR-517-5p** (0.700, p = 0.045). Using **miR-516-5p** (0.608, p = 0.146), **miR-518b** (0.550, p = 0.507), **miR-520a-5p** (0.495, p = 0.951), **miR-520h** (0.451, p = 0.538), and **miR-525-5p** (0.475, p = 0.755) prediction rules for preeclampsia had smaller areas under the curve and the predictive performance was not significant (Fig 2, Table 3). MiR-517-5p predicted preeclampsia with a sensitivity of 42.9%, a specificity of 86.2%, a PPV of 52.9%, and a NPV of 80.6%. First trimester screening based on the combination of all 6 tested circulating placental specific microRNAs (miR-516b-5p, miR-517-5p, miR-518b, miR-520a-5p, miR-520h, and miR-525-5p) was able to identify women at risk of developing preeclampsia with a sensitivity of 20.6%, a specificity of 90.8%, a PPV of 44.8%, and a NPV of 76.0%. Table 3 displays the predictive accuracy of maternal plasma concentrations of placental specific microRNAs in early pregnancy in the identification of preeclampsia using cut-offs derived from the ROC curves.

**Table 3:** Predictive accuracy of circulating C19MC microRNA biomarkers for preeclampsia.

miRNA	AUC	ROC curve	Sensitivity	Specificity	PPV	NPV	PLR	NLR	TP	TN	FP	FN
	(95% CI)	p-value	(95% CI)	(95% CI)								
**miR-517-5p**	0.700	0.045	42.9%	86.2%	52.9%	80.6%	3.107	0.663	9	50	8	12
	(0.497–0.792)		(24.5–63.5)	(74.7–93.0)								
**miR-516b-5p**	0.608	0.146	38.1%	84.5%	47.1%	79.0%	2.455	0.733	8	49	9	13
	(0.462–0.775)		(20.8–59.2)	(72.7–91.8)								
**miR-518b**	0.550	0.507	52.4%	63.8%	34.4%	78.7%	1.447	0.746	11	37	21	10
	(0.402–0.698)		(32.4–71.6)	(50.9–74.9)								
**miR-520a-5p**	0.495	0.951	23.8%	93.1%	55.6%	77.1%	3.452	0.818	5	54	4	16
	(0.337–0.653)		(10.4–45.6)	(83.0–97.7)								
**miR-520h**	0.451	0.538	14.3%	96.6%	60%	75.7%	4.143	0.888	3	56	2	18
	(0.294–0.607)		(4.3–35.7)	(87.4–99.7)								
**miR-525-5p**	0.475	0.755	14.3%	100%	100%	76.3%	ND	0.857	3	58	0	18
	(0.321–0.630)		(4.3–35.7)	(92.4–100.0)								
**6 C19MC microRNAs**	0.545	0.144	20.6%	90.8%	44.8%	76.0%	2.244	0.874	26	316	32	100
	(0.484–0.606)		(14.5–28.6)	(87.3–93.4)								

PPV; positive predictive value, NPV; negative predictive value, PLR; positive likelihood ratio, NLR; negative likelihood ratio, TP; true positive, TN; true negative, FP; false positive, FN; false negative

## Discussion

The current work is oriented towards novel insights into pathogenesis of preeclampsia and IUGR and potential improvement of diagnostical modalities. In order to unravel the causes of the failures in the maternal-fetal dialogue we focused on early pregnancy. To our knowledge, this is the first nested case control study from a longitudinal cohort reported to evaluate 1^st^ trimester maternal plasma concentrations of placental specific C19MC microRNAs present in pregnancies that went on to develop preeclampsia and IUGR. We were specifically interested in how the evolutionary conflict in parent-offspring relations in the placental bed is manifested in maternal circulation during early gestation. Genetic factors account for more than half of the incidence of preeclampsia, with maternal genes contributing more than fetal genes; couple effects can also occur because of the interaction between genes of the mother and the father [16, 17]. The C19MC cluster is imprinted, and exclusively expressed in the placenta from the paternally inherited allele [18]. C19MC microRNAs are expressed predominantly in placental trophoblasts during pregnancy, although they have also been detected in the testis, embryonic stem cells, and specific tumors [19–27]. The expression level of the C19MC cluster markedly increases in placental trophoblasts [28, 29] and maternal plasma from the first to the third trimester [30–34]. Our previous study demonstrated that upregulation of circulating C19MC microRNAs (miR-516b-5p, miR-517-5p, miR-520a-5p, miR-525-5p, and miR-526a) was associated with clinically established preeclampsia [8]. Furthermore, the dependence between the levels of plasma C19MC microRNAs and the pulsatility index in the middle cerebral artery (miR-516b-5p, miR-517-5p, miR-520a-5p, miR-525-5p, and miR-526a) and the values of the cerebroplacental ratio (miR-520a-5p, and miR-526a) was demonstrated in a cohort of pregnancies complicated with preeclampsia and/or fetal growth restriction [8]. The current study produced an interesting finding, i.e., up-regulation of circulating C19MC microRNAs (miR-517-5p, miR-518b, and miR-520h) is present in early pregnancy in those women destined to develop preeclampsia; while the other examined circulating C19MC microRNAs (miR-516b-5p, miR-520a-5p, and miR-525-5p) showed a trend toward up-regulation at 10 to 13 weeks of gestation in patients at risk of preeclampsia. Interestingly, up-regulation of miR-516b-5p, miR-517-5p, miR-520h, and miR-518b was also recently reported to be associated with a later occurrence of gestational hypertension [9].

In addition, the presence of higher first trimester plasma levels of miR-517-5p appears to be predictive of preeclampsia. The miR-517-5p biomarker alone had a predictive performance for preeclampsia with a sensitivity of 42.9%, a specificity of 86.2%, a PPV of 52.9%, and a NPV of 80.6%. There was no additive effect of using the combination of all examined circulating C19MC microRNAs to predict preeclampsia (sensitivity 20.6%, specificity 90.8%, a PPV of 44.8%, and a NPV of 76.0%).

Unfortunately, first trimester screening of women for C19MC microRNA biomarkers had no clinical utility relative to development of IUGR.

Individual maternal plasma/serum markers have not usually performed well as screening tests for preeclampsia and fetal growth restriction [35–45]. The predictive value of each biomarker is low; therefore, combined screening tests to assess the risk of preeclampsia and fetal growth restriction are currently used in practice [46–48]. Usually biochemical and biophysical tests are combined to assess placentation and maternal disease susceptibility [49]. In a proposed new approach to prenatal care, screening using a combination of maternal risk factors, mean arterial pressure, uterine artery Doppler, and maternal serum biomarkers (pregnancy-associated plasma protein-A and placental growth factor) can identify up to 95% of cases with early onset of preeclampsia for a false-positive rate of 10% [46, 47]. Another model including maternal characteristics, mean arterial pressure, uterine artery Doppler, placental growth factor, and soluble Fms-like tyrosine kinase-1 achieved an overall detection rate of 71.4% for fetal growth restriction, with a 10% false positive rate [48].

The addition of more efficient biomarkers for first trimester screening would certainly increase the predictive value of the diagnostic panel for preeclampsia.

Early prediction of severe pre-eclampsia would allow closer surveillance and earlier intervention to improve outcomes. It is likely that prospective treatments would need to start as early as possible, ideally before the 16^th^ week of gestation, in order to alter the pathogenesis [49].

Nevertheless, consecutive large scale studies are needed to assess sensitivity, specificity, and predictive value of the circulating miR-517-5p biomarker for preeclampsia. In addition, the diagnostic performance of the placental specific miR-517-5p biomarker, in relation to the severity of the disease with respect to clinical signs, requirements for the delivery, and Doppler ultrasound parameters, should be evaluated.

While the full repertoire of the biological action of C19MC microRNAs remains to be established, data from various expression studies of C19MC microRNAs imply a role for them in cell proliferation, self-renewal, angiogenesis, and particularly in pro-/anti-cancer activity [26, 28, 50]. In fact, there is not much research data about the function of miR-517-5p, miR-518b, and miR-520h in the literature. However, it is likely that similar mechanisms as those present in cancer development and tumor progression may also be in place during human placentation, which starts just after the implantation of the blastocyst into the epithelium of the uterus, and during vasculogenesis of the placental villi, which begins about the 5^th^ week of gestation. Since placental blood vessel formation happens in a relatively hypoxic environment until 10–12 weeks of gestation, there is a certain parallel with tumor biology. Hypoxia, which is a pivotal factor in tumor pathophysiology and a characteristic feature of locally advanced solid tumors, can promote tumor progression, since it is associated with restrained proliferation, differentiation, necrosis, and/or apoptosis [51].

It has been proposed that miR-518b may function as a tumor suppressor by targeting Rap1b [52], since Rap1b expression is negatively regulated by miR-518b. Rap1b is an isoform of Rap1, a small GTPase regulating adhesion, migration, polarity, differentiation, growth, and angiogenesis [53, 54]. Mir-518b has been shown to suppress cell proliferation by inducing apoptosis in tumor cells and invasion by targeting Rap1b [52]. Similarly, mir-520h targets ABCG2, which is highly expressed in several tumors, and alters cellular epigenetic programming to promote cell survival. Functional studies have indicated that loss of miR-520h expression is accompanied by subsequent activation of ABCG2 expression, which represent critical events in the invasion and migration of human pancreatic cancer cells [55]. Moreover, it has been shown that miR-520h functions as a potent suppressor of migration and invasion of human pancreatic cancer cells through down-regulation of ABCG2 expression [55]. MiR-520h is also crucial for DAPK2 (Death-associated protein kinase 2) regulation in breast cancer progression [56]. Mir-520h induced suppression of DAPK2 is associated with a poorer prognosis and lymph node metastasis in breast cancer patients [56].

Furthermore, Rg-3-induced overexpression of miR-520h results in the reduction of EphB2 and EphB4 and in subsequent angiosuppression [50]. Ephrins (Eph) mediate the critical steps of angiogenesis and vascular-network formation, including endothelial cell-to-endothelial/mesenchymal-cell interactions, cell adhesion to the extracellular matrix, cell proliferation, and migration [57]. Eph/ephrin signaling mechanisms may also correlate with VEGF-induced angiogenesis and VEGFR function in developmental and tumor angiogenesis [50, 58, 59]. Mir-520h induced down-regulation of EphB2 and EphB4 in endothelial cells could lead to inhibition of VEGFR-2 expression and angiosuppression [50]. In addition, silencing of CXCR4, a rhodopsin-like G-protein-coupled receptor that selectively binds CXCL12 chemokine, by miR-520h has been shown to successfully block invasion and metastasis of cancer cells [60]. The binding of CXCL12 to CXCR4 activates various signaling pathways such as calcium influx, phosphoinositide 3 (PI3) kinase, mitogen-activated protein (MAP) kinase, Src kinase and Rho [61]. Additionally, altered CXCR4 expression results in tumor growth, angiogenesis, invasion, and metastasis [62, 63].

Unfortunately, functional roles for miR-517-5p have not yet been demonstrated. Available prediction algorithms usually predict hundreds of potential target genes for a single microRNA, but often generate false-positive candidates [64]. We reported a list of predicted target genes of differently expressed C19MC microRNAs in pregnancy-related complications, in relation to immune system and the inflammatory response, in our previous study dedicated to the expression profile of C19MC microRNAs in placental tissues [3].

Different C19MC microRNA expression profiles in different cell types within villous tissue and in different areas of placental tissues were documented. The expression of C19MC microRNAs has been observed at least in first-trimester and full-term placental tissues [29, 65], human first and third trimester trophoblast cell lines, ACH-3P and AC1-M59 [66], and placenta-derived stromal cells [67]. In our initial study, we have observed the presence of all 16 tested C19MC microRNAs (miR-512-5p, miR-515-5p, miR-224, miR-516-5p, miR-517*, miR-136, miR-518f*, miR-519a, miR-519d, miR-519e, miR-520a*, miR-520h, miR-524-5p, miR-525, miR-526a and miR-526b) on the fetal side of the placenta [31]. In addition, the set of microRNAs (miR-517c, miR-518a, miR-519d, and miR-520h) forming a cluster on chromosome 19q13 was observed to be expressed in umbilical cord blood CD34+ cells [68]. Gu et al. [69] previously showed that the optimal solution was to test the microRNA expression profile in whole villous tissue containing cytotrophoblasts, syncytiotrophoblasts, mesenchymal/stromal cells, villous core fetal vessel endothelium, etc. The microRNA expression profile in whole villous tissue closely resembles microRNA expression without disruption of tissue integrity in an in vivo situation [69]. Therefore, we previously analyzed C19MC microRNA gene expression in whole villous tissue, but in the specific area of the central cotyledon zone, where the umbilical cord inserts into the chorionic plate. Although, we did not specifically examine the localization of C19MC microRNAs within villous tissue, our data suggested that pregnancy-related complications were associated with alterations in placental microRNA expression [3]. The retrospective study design enabled us to test diverse biological material of equal patients (i.e., placental tissues, maternal plasma samples collected at 10 to 13 weeks of gestation, and during the onset of pregnancy-related complications). Nevertheless, in contrast to maternal circulation, down-regulation of C19MC microRNAs was found in placental tissues derived from patients with (1) gestational hypertension (miR-517-5p, miR-519d, miR-520a-5p and miR-525), (2) fetal growth restriction (miR-517-5p, miR-518f-5p, miR-519a, miR-519d, miR-520a-5p and miR-525), and (3) clinically established preeclampsia (miR-515-5p, miR-517-5p, miR-518b, miR-518f-5p, miR-519a, miR-519d, miR-520a-5p, miR-520h, miR-524-5p, miR-525 and miR-526a) [3]. Other independent studies have also observed decreased expression at least of some C19MC microRNAs in preeclamptic placentas (miR-518b and miR-525, [70]) or FGR (miR-515-5p, miR-518b, miR-519d, miR-520h, and miR-526b, [71]). Although, C19MC microRNAs were found to be down-regulated around the central cotyledon in patients with clinically established preeclampsia [3], they can be up-regulated in other areas of placenta tissues, as has been shown in several independent studies. For example, Xu et al. [72] observed up-regulated expression of miR-518b in basal plates of severe preeclamptic placentas and Ishibashi et al. [73] revealed up-regulation of miR-525, miR-518f-5p, miR-526b and miR-519e-5p in preeclamptic placentas, but no details regarding the sampling location of placental tissue was provided in the study. We believe that variable levels of circulating C19MC microRNAs in patients affected with pregnancy-related complications such as gestational hypertension, preeclampsia, and FGR can be influenced by compilations stemming from several factors. At the very least, an expression of particular circulating C19MC microRNA is represented by the total sum of expression of this particular C19MC microRNA in individual cells located in different areas of placenta, which actively secrete exosomes mediating intercellular communication, currently undergo apoptosis, or release placental debris into the maternal circulation. It has been clearly demonstrated that the establishment of a balance between trophoblast proliferation and apoptosis is crucial during normal placental development [74]. Both aging syncytiotrophoblasts and extravillous cytotrophoblasts undergo apoptosis [75–77]. Therefore, extracellular nucleic acids (DNA, mRNA, and microRNAs) of both fetal and placental origin, packed into trophoblast-derived apoptotic bodies, can be detected in the maternal circulation during the normal course of gestation [78]. In addition, human chorionic villi can secrete microRNAs extracellularly via exosomes, which can enter the maternal circulation [30]. Several recent studies have showed increasing levels of circulating nucleic acids (fetal DNA, placental specific mRNA transcripts, and C19MC microRNAs) with advancing gestation, which reflects the growth of the placenta [7, 31, 79–82]. Both absolute and relative quantification approaches have revealed significant increases, over time, in extracellular placental specific C19MC microRNA levels (miR-516-5p, miR-517-5p, miR-518b, miR-520a-5p, miR-520h, miR-525 and miR-526a) in women with normally progressing pregnancies [7]. The highest concentrations and expression of circulating C19MC microRNAs have been observed during the third trimester (36^th^ week of gestation), whereas differences have been found between the first (12^th^ week of gestation) and the second (25^th^ week of gestation) trimesters only in 4 out of 7 tested placenta-specific microRNAs (miR-516-5p, miR-517-5p, miR-518b, and miR-520h) [7].

Chronic placental hypoxia is one of the root causes of placental insufficiencies that result in preeclampsia and maternal hypertension [83]. One of the most probable hypothesis to describe the etiology of preeclampsia is based on a failure of extravillous trophoblasts to invade the uterine spiral arteries in the placental bed. This results in placental hypoxia and subsequent damage to villous trophoblasts [83]. Hypoxic environment induces excessive trophoblast cell death and increased shedding of placenta debris into the maternal circulation. Increased apoptosis has been observed in extravillous trophoblasts of placentas; although, mainly in pregnancies complicated by preeclampsia [84]. As a result, placental insufficiency related pregnancy complications (preeclampsia) have also been associated with abnormal levels of extracellular fetal DNA, mRNA transcripts, and circulating C19MC microRNAs (miR-516b-5p, miR-517-5p, miR-520a-5p, miR-525-5p, and miR-526a) [8, 77, 85].

Similarly, increased extravillous trophoblast sensitivity to apoptotic signals in the first trimester of gestation, due to reduced NO synthesis in pregnancies at higher risk of developing preeclampsia, can cause higher levels of circulating C19MC microRNAs. Whitley et al. [86] showed that first trimester extravillous trophoblasts from pregnancies with high uterine artery resistance were inherently more sensitive to apoptotic stimuli, which can be associated with reduced remodeling of the maternal spiral arteries.

Fetal growth restriction is a complex disease, resulting from an array of diverse etiologies, which is characterized by a failure of the fetus to reach its growth potential. Recent studies have demonstrated that plasma levels of the majority studied microRNAs were not significantly different in women with FGR [87], despite the fact that their expression has been shown to be altered by hypoxia in trophoblasts under *in vitro* conditions [88]. Analogous to our previous and current study [8, 71] also observed reduced expression of certain C19MC microRNAs in placentas of FGR patients, but circulating levels of these substances in maternal plasma showed no significant differences between FGR and uncomplicated pregnancies. The most likely explanation is a hypothesis presented by Huppertz et al. [89] suggesting that during intra-uterine growth restriction, placental oxygenation may be increased rather than decreased, and therefore at least some IUGR patients have no signs of trophoblast injury and consequential placental dysfunction, which can lead to increased data variability for IUGR pregnancies [88].

In conclusion, C19MC microRNAs play a role in the pathogenesis of pregnancy-related complications. Our current and previous studies demonstrated for the first time that circulating C19MC microRNAs are dysregulated in maternal circulation early in the pregnancy and might play a role in the inducement of gestational hypertension and preeclampsia.

## Supporting information

S1 Table S1 Table. (XLS)
